# Sprints, Decelerations and Turns Most Commonly Precede Goals in Soccer: Analysis of 6 FIFA World Cups

**DOI:** 10.1002/ejsc.70085

**Published:** 2025-11-23

**Authors:** Lorcan Daly, Patrick Caulfield, David Martínez‐Hernández

**Affiliations:** ^1^ Department of Sport and Health Sciences Technological University of the Shannon Athlone Ireland; ^2^ SHE Research Group Technological University of the Shannon Athlone Ireland; ^3^ Department of Physical Education and Sport Sciences University of Limerick Limerick Ireland; ^4^ Sport and Human Performance Research Centre University of Limerick Limerick Ireland; ^5^ Directorate of Sport Exercise and Physiotherapy University of Salford Salford UK

**Keywords:** match intensity, movement analysis, physical fitness, soccer performance, turning

## Abstract

This study investigated actions preceding goals in male and female players across 6 FIFA World Cups. This is the first longitudinal, multi‐sex analysis of goal actions using world‐class data, extending validity and findings beyond the current evidence base (domestic, single‐sex studies). In total, 2995 actions preceding goals from open‐play were analysed across the last 6 men's’ and women's FIFA world cups (2014–2023) using the modified Bloomfield method. Additionally, possible longitudinal (tournament cycle), sex, and role‐based (i.e., scorer vs. assister) differences were examined using Bayesian and chi‐squared analyses. Linear advancing movements (≈41%), particularly sprinting, were the most prevalent actions leading to goals, followed by deceleration (≈22%) and turns (≈19%) (Cramer's V = 0.27–0.38; *p* < 0.05). Longitudinal, between‐sex and between‐role (i.e., scorer vs. assister) differences were predominantly minimal for movement types (Bayes Factors [BF_10_] < 0.01; Cramer's V = 0.02–0.06; *p* > 0.05). Sprinting preceded goals more prevalently for males (43.4%) and scorers (43.2%), when compared with females (39.0%) and assisters (39.1%), respectively (Cramer's V = 0.04–0.10; *p* < 0.05). Female players performed significantly greater proportions of actions at high intensity versus males (53.0 vs. 47.9%; BF_10_ = 38.7; Cramer's V = 0.369; *p* < 0.05), and the 2022/2023 cycle had lower proportion of actions at high intensity compared to earlier cycles (46.6% vs. 52.3%–52.5%; BF_10_ = 0.019; Cramer's V = 0.06; *p* < 0.05). This analysis highlights the importance of sprinting, decelerating, and turning for goal‐scoring. Therefore, enhancing players' physiological and mechanical reserves to undertake these actions, as and when required during match‐play, appears prudent. Further, analysts/coaches may apply this information to identify decisive goal‐scoring actions and design targeted training drills accordingly.

## Highlights

- This is the first longitudinal, multi‐sex and international‐calibre analysis of the actions preceding goals during the recent 6 FIFA World Cup tournaments (2014–2023).
- Sprints, decelerations, and turns were the most common actions preceding goals from open play, with minimal differences detected across sex, player role, or tournament cycle.
- Accordingly, improving players' sprint speed, deceleration, and turning abilities appear critical strategies for practitioners tasked with increasing players' capacity to generate and/or defend against goal‐scoring chances, respectively

## 1 Introduction

Soccer is an intermittent field‐based team sport where successful match‐play is governed by numerous factors, such as technical abilities, tactical knowledge, contextual variables, cognition, physical conditioning and in‐game work rates (Augusto et al. 2023; Paul et al. 2015). Among these, high intensity actions like sprints, decelerations and changes of direction are documented to contribute to goal scoring (Martínez‐Hernández et al. 2024). During competition, players execute challenging technical skills, regularly contest for possession and frequently adapt on‐field positioning according to dynamic tactical requirements (Aquino et al. 2020; Augusto et al. 2023; González‐Ródenas et al. 2019). Notwithstanding the significant technical‐tactical proficiency necessary for high performance, the unpredictable and rapid nature of the game undergirds the importance of an array of metabolic, neuromuscular and mechanical attributes (Aquino et al. 2020; Daly et al. 2023).

Although beneficial for many insights, cataloguing players' numerical workload measures, such as those collected via microtechnology devices (e.g., global or local positioning systems), may lack contextual sensitivity when seeking to identify critical match actions such as successful turnovers, counterattacks, and goals (Paul et al. 2015; Ponce‐Bordón et al. 2022). Regarding the latter, research has implicated the intricate nature of effectively generating attacking opportunities and scoring goals (Schulze et al. 2022). Tactical and situational factors such as inter‐team balance (i.e., the organisation of defending players relative to opposing attackers), gaining possession close to the opposition's goal (Gonzalez‐Rodenas et al. 2020), and the proximity and/or number of attacking players between the goal and ball (Schulze et al. 2022) were all demonstrated to influence successful scoring opportunities (Schulze et al. 2018).

In order to create scoring opportunities from play, rigorous periods of elevated running demands, such as during rapid counterattacks or attackers creating separation from defenders, have been observed to be associated with goals (Schulze et al. 2022). By extension, attacking players with highly developed physical conditioning attributes (e.g., including the ability to change pace or direction quickly, reach high running velocities, and sustain repeated high‐intensity efforts) are likely well positioned to disrupt opponents' defencive structures and creating space for goal‐scoring chances (Martínez‐Hernández et al. 2023; Schulze et al. 2022; Schulze et al. 2018). These capacities are likely underpinned by many physiologic characteristics, spanning neuromuscular, mechanical, metabolic and cardiovascular attributes (Daly et al. 2023; Vigh‐Larsen et al. 2024). Consequently, it may then be conceivable that high‐intensity actions, and in turn the physiological attributes underpinning their performance, may be important contributors to the creation of successful goal scoring opportunities (Martínez‐Hernández and Jones 2024; Martínez‐Hernández et al. 2023). However, work examining the specific physical actions preceding goals remains in its infancy (Faude et al. 2012; Martínez‐Hernández and Jones 2024; Martínez‐Hernández et al. 2023, 2024). Therefore, many important questions remain, such as (i) what occurs at the top level of international competition? (ii) are there differences according to biological sex? (iii) as the game rapidly evolves and becomes ever more intensive (Harper et al. 2021), are the physical determinants of goal scoring changing in parallel? In order to adequately resolve these uncertainties, analyses using large sample sizes are required.

Remarkably, the empirical evidence to date which has analysed the physical actions which determine goal scoring in soccer is limited to four studies (Faude et al. 2012; Martínez‐Hernández et al. 2023, 2024), while a recent study using GPS reported elevated high‐speed running (> 24 km·h^−1^), but not other physical outputs, in the 5 min preceding goals versus match average (Asian‐Clemente et al. 2025). Collectively, these works have provided excellent insight, suggesting that intensive actions such as sprinting, decelerating and turning are commonly undertaken by attacking players in the lead up to goals (Faude et al. 2012; Martínez‐Hernández and Jones 2024; Martínez‐Hernández et al. 2023, 2024). Nevertheless, these studies are each confined to one season in a European league, namely the English premier league [EPL], women's super league [WSL] and the German Bundesliga (Faude et al. 2012; Martínez‐Hernández and Jones 2024; Martínez‐Hernández et al. 2023, 2024), respectively. This precludes assessment of longitudinal changes or possible international variations, thereby rendering an evidence base with important limitations.

Given (i) the outlined gaps evident in the available literature, namely their restriction to single‐sex, domestic national league contexts and single‐season temporal scope, and (ii) broader concerns regarding the replicability of findings in sport science (Mesquida et al. 2022) there is a clear and urgent need to validate preliminary observations. Therefore, examining the actions leading to goals in a larger, longitudinal sample of both men's and women's competitions at the top international level of the sport would provide critical novel insights. Such work would offer a more robust, ecologically valid understanding of the physical actions preceding goals, serving to (i) inform training programme design, enabling the targeted development of physical conditioning attributes which underpin key goal scoring actions, (ii) identify if there are any longitudinal trends occurring in goals scoring events and (iii) deliver biological sex specific considerations for these factors, if such differences were uncovered. Therefore, this study aims to address the aforementioned knowledge gaps by examining and comparing the actions preceding all goals from play in recent women's (2015, 2019 and 2023) and men's (2014, 2018 and 2022) FIFA World Cups.

## 2 Materials and Methods

### 2.1 Experimental Approach to the Problem

This study analysed the movement patterns preceding 649 open‐play goals from 6 FIFA World Cups (3 men's, 3 women's). Video analysis focussed on the final three movements of the scorer and assister preceding goals, categorized using a modified Bloomfield Movement Classification (mBMC) (Bloomfield et al. 2004) with added modifiers for intensity, direction and ball involvement. By examining open‐play scenarios exclusively, the study aimed to provide actionable insights into the physical and tactical demands of goal‐scoring actions. The analysis also assessed whether longitudinal changes or differences based on biological sex were evident in the actions leading to goals.

### 2.2 Procedures

As the data were fully accessible to the public, institutional ethical approval was deemed to be not required. Actions preceding all goals from open play during the women's (2015, 2019, and 2023) and men's (2014, 2018 and 2022) FIFA World Cups were analysed through video analysis. Motion analysis was performed for attacking players involved in the goals. The attacking player that scored the goal was termed ‘‘scorer’’ and the player performing the assist to the scorer was termed 'assister’.

A single investigator conducted the analysis and coding of the goal actions, with no time restrictions for the analysis of each movement. The coding was carried out by the coauthor using a computerized notation system within a customized Excel spreadsheet (Office 365 ProPlus). Analysis was performed for the last three movements before the pass executed by the assister, and the last three movements before shot by the scorer, respectively. The decision to focus on the last three passes was made to provide a structured, consistent framework for analysis, ensuring practical insights while managing data complexity and a granular examination of actions in immediate proximity to goals (Faude et al. 2012; Martínez‐Hernández and Jones 2024; Martínez‐Hernández et al. 2023, 2024).

This dataset, drawn from 649 goals from play across 6 men's and women's FIFA World Cups, ensures a broad and robust platform for analysis. While previous studies analysed a larger sample of 769 (Martínez‐Hernández et al. 2023) and 1025 (Martínez‐Hernández and Jones 2024) goals respectively, these were limited to national‐level competitions, potentially reducing the diversity of tactical and physical contexts. Additionally, the remaining literature analysed smaller samples (Faude et al. 2012; Martínez‐Hernández et al. 2024), each limited to single domestic competitions. By focussing on the final three movements, we aimed to maintain a precise and manageable scope that highlights the most decisive actions while fully leveraging the breadth and diversity of the dataset. This approach elicits robust and actionable insights without overcomplicating the analysis or relevance of the findings. However, it is important to acknowledge that the choice of three movements as a cutoff point is inherently arbitrary, similar to the previously used cut offs (Faude et al. 2012; Martínez‐Hernández and Jones 2024; Martínez‐Hernández et al. 2023, 2024) as any such limit would be. Future research should therefore consider exploring a broader range of actions, even if this may dilute the immediate proximity to goals, or analysing a smaller subset of actions across an even larger database to uncover additional insights. In support of such endeavours, the present dataset is open‐access and can be used for reanalysis (https://shorturl.at/OWk2X).

Motion analysis for the assister started just before receiving the ball from a teammate or when this regained ball possession from the opposition. For the scoring player motion analysis started when the ball was passed from the assister and finished when the scorer shot to the goal. Goals from set‐pieces, own goals, and unintended or rebound goals were excluded to focus exclusively on open‐play scenarios, as set‐pieces involve distinct dynamics, and may therefore warrant separate investigation (Faude et al. 2012). This study focussed on open‐play scenarios only with the aim to provide a more homogeneous sample and yield more concrete findings within this specific context.

**FIGURE 1:**
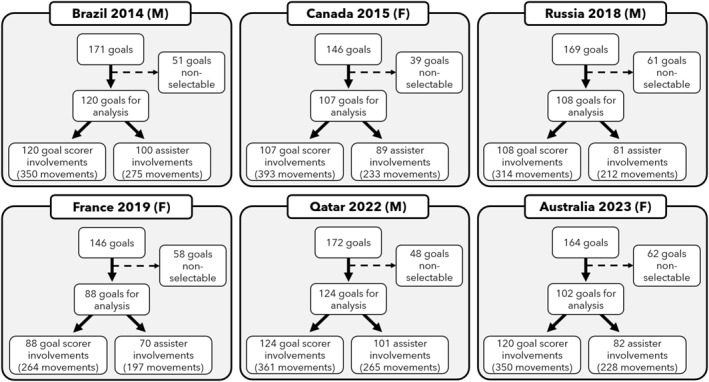
Flow chart for each world cup for the goals selected for analysis and total involvements for the scorer and assister. non‐selectable goals include own goals, rebound, penalties, indirect free kicks, direct free kicks, corners and throw ins. M, male; F, Female.

The movements leading up to the goals were analysed using a modified version of the mBMC (Bloomfield et al. 2004) as described by Martínez‐Hernández et al. (Martínez‐Hernández et al. 2023, 2024). Of note, the specific definitions of all movements/intensities and other moderators can be seen in Supporting Information S1: Tables S1 and S2. Similar to the aforementioned works from Martínez‐Hernández et al., the following modifiers were added/adapted, extending from the original model (Bloomfield et al. 2004): intensity (low, medium and high intensity), direction and ball involvement. The following movements were considered for analysis: linear advancing motion (walk and jog [little effort; ‘low intensity’], run [some to great effort; ‘medium intensity’] and sprint [maximal effort; ‘high intensity’]), lateral advancing motion (crossover and shuffle), turn, deceleration, impact, stand still, jump, land and fall and get up. While the ball modifier was introduced in every movement, the direction modifier was applied to linear advancing motion, deceleration, turn and skip movements. In addition, linear advancing motion (sprint, run, jog, walk), deceleration, turn (to rotate whilst standing, decelerating or accelerating/sprinting), change in angle run (cut [Path change of less than 45° with this involving little or no previous deceleration] and arc [Player (often leaning to one side) moving in a semi‐circular direction] run) and lateral advancing motion (shuffle and crossover) had an intensity modifier as per (Bloomfield et al. 2004). Of note, ‘Arc’ run movement is described elsewhere in the literature as a ‘curved run’ or ‘curvilinear sprint’ (e.g. (Solleiro‐Duran et al. 2025),), however we retain the term ‘arc run’ for consistency with the original Bloomfield coding framework. Additionally, in this coding framework, movements such as ‘Turn’ and ‘Cut’ are classified as discrete technical actions. While these movements involve directional reorientation, they are not synonymous with the broader construct of ‘change of direction’ (COD), which in sports science typically refers to an athlete's physical ability to rapidly decelerate and re‐accelerate when changing direction (Nygaard Falch et al. 2019). In this sense, whilst a movement involving a turn would generally be considered as a COD, other movements that do not include a turn can also be defined as a COD. For example, when performing a sharp cut or when shuffling to one side, followed by a lateral deceleration and a lateral shuffle to the opposite side (Martínez‐Hernández and Jones 2024). Figure 1 provides an overview of the goals included in the analysis.

### 2.3 Statistics

Data were examined using Chi‐squared tests in SPSS for Windows version 22.0 (SPSS Inc., Chicago, IL) to examine differences between variables. The prevalence and intensity of movement occurrences preceding goals were analysed across the 6 most recent men's and women's world cups. Herein, possible differences in movement prevalence and intensity for the different competition cycles (2014/2015, 2018/2019 and 2022/2023), biological sexes and player roles (assister and scorer) were examined.

To obtain inter‐rater reliability, videos corresponding to 10 clips of each movement were analysed by two investigators. To assess intra‐rater reliability, the same co‐author analysed 100 movement clips. Inter‐rater and intra‐rater reliability were assessed using unweighted Cohen's Kappa statistics, calculated across key movement dimensions (movement type, intensity, direction, and ball interaction). Kappa values were interpreted based on standard benchmarks (e.g., < 0.20; slight; 0.21–0.40; fair; 0.41–0.60; moderate; 0.61–0.80; substantial; > 0.81; almost perfect agreement) (Cohen 1992). The normality of the data was assessed using the Kolmogorov‐Smirnov test. Strength of association was reported using Cramer's V, with interpretation based on the degrees of freedom, as recommended Cohen (1992). Statistical significance was accepted at an alpha level of *p* < 0.05.

Additional Bayesian contingency table analyses were conducted in JASP (version 0.19). These analyses were performed to provide Bayes Factors (BF_10_) quantifying the strength of evidence for or against associations between (i) key categorical variables, hereinafter: movement type and intensity, or intensity and role and (ii) player role, biological sex and competition. Bayesian methods offer a continuous measure of evidence, allowing for the evaluation of the null hypothesis. Together, the chi‐squared and Bayesian contingency analyses provide both comparability with existent literature (Martínez‐Hernández and Jones 2024; Martínez‐Hernández et al. 2023, 2024) and may support stronger inferential conclusions by combining traditional significance testing with a graded measure of evidence strength. BF_10_ were interpreted in line with the classification scheme of Lee and Wagenmakers (2014): (i) *for the null hypothesis (H*_*0*_ *);* BF_10_ = 0.33–1 indicates anecdotal evidence; 0.10–0.33, moderate evidence; 0.03–0.10, strong evidence; 0.01–0.03, very strong evidence; and < 0.01, extreme evidence; (ii) *for the alternative hypothesis (H*_1_ *),* BF_10_ = 1–3 indicates anecdotal evidence; 3–10, moderate evidence; 10–30, strong evidence; 30–100, very strong evidence; and > 100, extreme evidence.

## 3 Results

**FIGURE 2:**
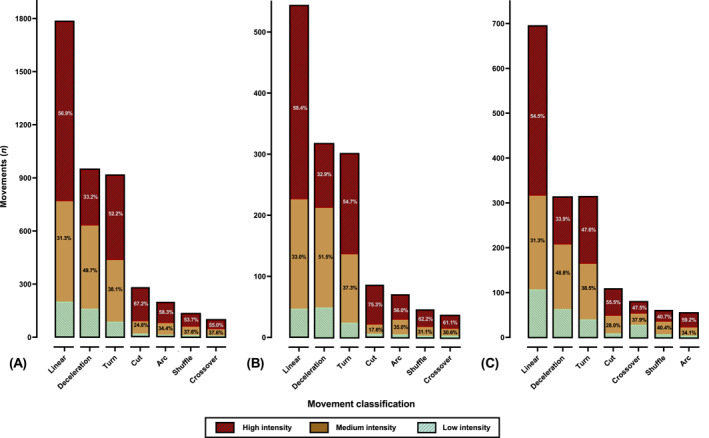
Movement classification and intensity prevalence for (A) overall, (B) female and (C) male world cup goals. Linear: linear advancing movement.

**FIGURE 3:**
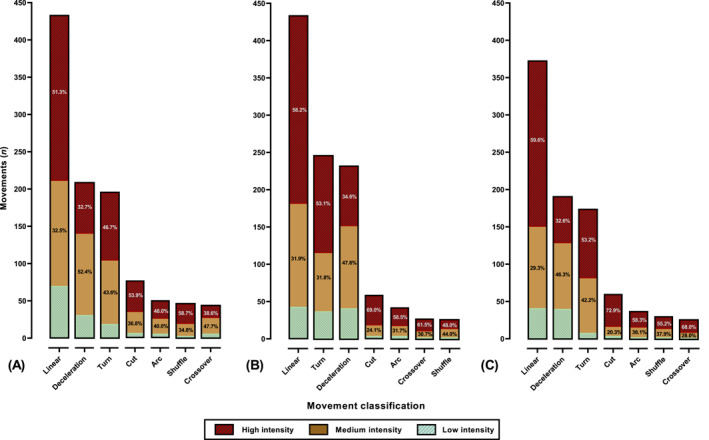
Movement classification and intensity prevalence for (A) 2023/2022, (B) 2019/2018 and (C) 2015/2014 world cup goals. Linear: linear advancing movement.

**FIGURE 4:**
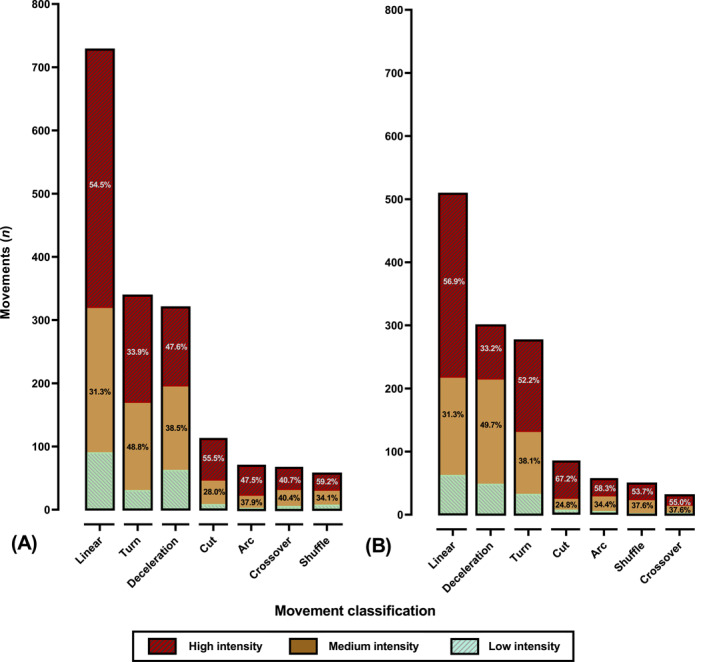
Movement classification and intensity prevalence for the (A) scorer and (B) assister during world cup goals. Linear: linear advancing movement.

**FIGURE 5:**
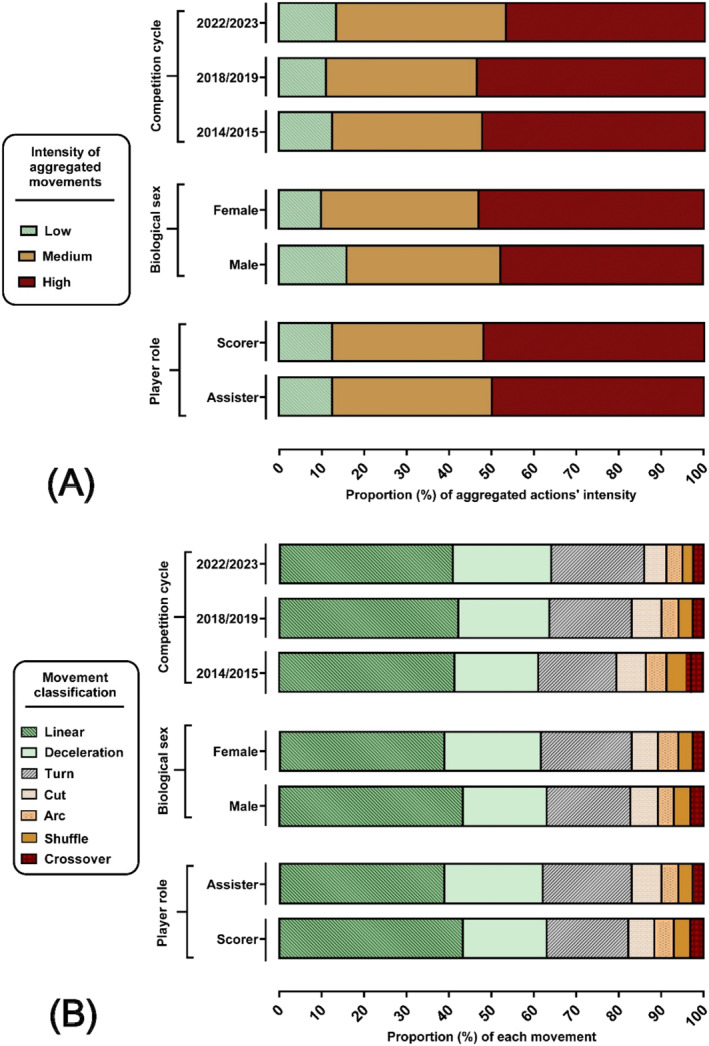
Proportional (A) intensity (%) and (B) movement distribution for each population group.

The Cohen's Kappa demonstrated high inter‐ (movement agreement: *k* = 0.864 and intensity agreement: *k* = 0.762) and intra‐rater (movement agreement: *k* = 0.916 and intensity agreement: *k* = 0.895) levels of agreement. The present data were non‐normally distributed (*p* < 0.05). Data are presented as follows: (i) overall and by sex in Figure 2, (ii) competition cycle in Figure 3, (iii) player role in Figure 4, and (iv) proportional (%) intensity and movement distribution for all comparisons in Figure 5.

### 3.1 Total Frequency and Percentages of Movements

In total, 2995 movements were examined in the 6 most recent FIFA World Cups analysed (968 total goals with 649 goals selectable for analysis). Within men's competitions, 579 movements were analysed in the 2014 (120 goals analysed), 463 in the 2018 (108 goals analysed) and in 559 the 2022 World Cup (124 goals analysed). With regard to women's world cups, 480 movements were analysed in the 2015 (107 goals analysed), 421 in the 2019 (88 goals analysed) and 493 in the 2023 World Cup (102 goals analysed) (Figure 1).

### 3.2 Movement Classification and Intensity Prevalence

Overall, linear advancing motion occurred most prevalently at high intensity (sprinting; x^2^_(2)_ = 537.22, *p* < 0.01, Cramer's V = 0.380), indicating a large effect. Linear advancing motion at high intensity (sprinting) was performed in similar percentages when comparing between sexes (x^2^_(1)_ = 1.83, *p* = 0.17, Cramer's V = 0.038; medium effect) roles (x^2^_(1)_ = 0.15, *p* = 0.7, Cramer's V = 0.009; small effect) and cycles (x^2^_(2)_ = 1.21, *p* = 0.33, Cramer's V = 0.069; small effect). Decelerations occurred predominantly at medium intensity (x^2^_(2)_ = 136.02, *p* < 0.01, Cramer's V = 0.268; medium effect), with no differences between sexes (x^2^_(1)_ = 1.96, *p* = 0.16, Cramer's V = 0.055; small effect), and cycles (x^2^_(2)_ = 1.68, *p* = 0.43, Cramer's V = 0.118; small effect), whilst assisters performed higher proportion of decelerations at medium intensity versus. scorer (x^2^_(1)_ = 12.02, *p* < 0.001, Cramer's V = 0.139; medium effect). Turns showed to be most commonly performed at high intensity (x^2^_(1)_ = 238.79, *p* < 0.001, Cramer's V = 0.369; medium effect) with no differences between sexes (x^2^_(1)_ = 5.89, *p* = 0.53, Cramer's V = 0.070; small effect), cycles (x^2^_(2)_ = 2.21, *p* = 0.33, Cramer's V = 0.06; small effect) and roles (x^2^_(1)_ = 0.89, *p* = 0.34, Cramer's V = 0.038; small effect). Other less frequent movements, such as lateral movements and change in angle runs, showed higher intensity variability but generally, movements were predominantly performed at high intensity (Figure 2, 3, 4, 5).

### 3.3 Intensity of Aggregated Movements

The proportion of high‐intensity actions showed no differences between roles (assister vs. scorer) (x^2^_(1)_ = 1.28, *p* < 0.26, Cramer's V = 0.020; small effect) but was different between competition cycles (2014/2015 vs. 2018/2019 vs. 2022/2023) and sex (male vs. female). In this regard, female players performed significantly greater proportions of actions at high intensity (x^2^_(1)_ = 5.82, *p* < 0.001, Cramer's V = 0.369; medium effect) whilst the 2022/2023 cycle had lower proportion of actions at high intensity compared to 2014/2015 and 2018/2019 cycles (x^2^_(2)_ = 11.38, *p* < 0.004, Cramer's V = 0.060; small effect) (Figure 4).

Regarding the Bayesian analysis, intensity levels appeared relatively consistent across World Cup cycles, with only minor variations (BF_10_ = 0.019), suggesting moderate evidence in favour of the null hypothesis of no meaningful difference. A significant difference was found in movement intensity between female and male players (BF_10_ = 38.73), indicating strong evidence for sex‐based effects. Here, high‐intensity actions were more common in female players (53.3%) than males (48.9%), whilst males performed more low‐intensity movements (14.9% vs. 9.8%). No meaningful differences were found in movement intensity between scorers and assisters (BF_10_ = 0.007), representing strong support for the null hypothesis.

### 3.4 Movement Proportions

Chi‐squared analysis revealed significant differences in movement proportions (x^2^_(6)_ = 2985.38, *p* < 0.001, Cramer's V = 0.300; large effect). Overall, the most common movement preceding goals in World Cups was a linear advancing motion, followed by deceleration and turn, with no significant differences between these last two movements (x^2^_(1)_ = 0.26, *p* = 0.61, Cramer's V = 0.006; small effect). Thereafter, next in proportion were change in angle runs (cuts and arc runs), followed by lateral advancing movements.

Linear advancing motion was performed in higher proportion in males versus. females (x^2^_(1)_ = 28.35, *p* < 0.01, Cramer's V = 0.097; small effect) and in scorers versus. assisters (x^2^_(1)_ = 3.97, *p* = 0.046, Cramer's V = 0.041; small effect), respectively. In contrast. No significant differences were found according to competition cycles (x^2^_(2)_ = 1.46, *p* = 0.48, Cramer's V = 0.022; small effect). Decelerations were significantly more prevalent in females compared to males (x^2^_(1)_ = 4.18, *p* = 0.040, Cramer's V = 0.038; small effect) and in assister versus. scorer (x^2^_(1)_ = 6.98, *p* < 0.01, Cramer's V = 0.049; small effect), whilst no significant difference was found between the different competition cycles (x^2^_(2)_ = 1.51, *p* = 0.47, Cramer's V = 0.022; small effect). On the other hand, there were no significant differences in the proportion of turns performed when compared between sexes (x^2^_(1)_ = 1.66, *p* = 0.19, Cramer's V = 0.024; small effect) competition cycles (x^2^_(2)_ = 10.03, *p* < 0.01, Cramer's V = 0.057; small effect) and roles (x^2^_(1)_ = 0.62, *p* = 0.43, Cramer's V = 0.057; small effect). On the other hand, more turns were performed in the 2014/2015 cycle versus. the 2022/2023 cycle (x^2^_(1)_ = 9.33, *p* < 0.01, Cramer's V = 0.067; small effect). In addition, cut, arc run, shuffle and crossover all showed comparable proportions when compared between sexes, roles and cycles, except for crossover. In this sense, more crossovers were performed in the 2022/2023 cycle versus. the 2014/2015 cycle (x^2^_(1)_ = 4.39, *p* < 0.03, Cramer's V = 0.048; small effect).

Bayesian analysis (BF_10_ = 0.007) provided strong evidence for the null hypothesis, indicating that movement types prior to goals are consistent between scorers and assisters. Bayesian analysis comparing movement types between female and male players resulted in a Bayes Factor of BF_10_ < 0.001, strongly supporting the null hypothesis and implicating highly consistent movement patterns across sexes. Bayesian analysis of movement type distributions across the world cup cycles produced a Bayes Factor of BF_10_ < 0.0001, strongly supporting the null hypothesis and indicating high overall movement type consistency across tournament cycles.

## 4 Discussion

This study examined the movement patterns occurring during goal‐scoring situations in the 6 most recent men's and women's FIFA world cups. Principal among the findings was that linear advancing movements were the most prevalent actions preceding goals, followed by decelerations, turns, change in angle runs and lateral advancing actions. Despite well‐documented differences in match‐play demands between men's and women's football (Modric et al. 2023; Riboli et al. 2024), as well as progressive annual increases in match intensity (Harper et al. 2021), longitudinal and biological sex differences in the actions leading to goals were minimal (Figures 1, 2, and 3). This inference is supported by the small effect sizes in most comparisons, and strong Bayesian evidence for the null hypothesis regarding longitudinal and biological sex comparisons. Indeed, the most recent world cup cycle had a lower proportion of movements at high intensity when compared with the two preceding cycles. Intensity levels appeared relatively consistent across World Cup cycles, with only minor variations, suggesting moderate evidence in favour of the null hypothesis of no meaningful difference. Finally, when comparing player roles, scorers and assisters exhibited similar proportions of actions at comparable intensities (Figures 4 and 5).

The current findings align with earlier studies using the same modified Bloomfield method (Martínez‐Hernández and Jones 2024; Martínez‐Hernández et al. 2023, 2024), thereby implying (i) possibly good levels of methodological replicability and (ii) generalizability of the results to different soccer populations. The presence of consistent findings across datasets, roles, and competitions, as evidenced by negligible Cramér's V values and strong Bayesian support for the null hypothesis (i.e., distributional similarity), may further reinforce the reliability of the method and results. Additionally, the similarity of actions observed between male and female players and scorers and assisters, across six World Cups, suggests that the proportion and intensity of actions leading to goals follow relatively consistent trends (Figures 4 and 5).

The present observation that linear advancing motions (principally sprinting), are prevalently associated with successful goals may suggest a potential relevance of acceleration and/or high peak speeds in offensive play, although the lack of specific metrics, such as sprint distance or speed, renders this premise tentative (Faude et al. 2012; Gamble et al. 2024; Ward et al. 2024). The importance of developing acceleration and peak speed may be reinforced by accumulating evidence that (i) players of a superior standard display greater running speeds (Koudellis et al. 2025), (ii) match‐play is becoming more intensive (Harper et al. 2021), (iii) running speed and match workload indices are positively associated (Daly et al. 2023) and (iv) effective speed development training is associated with reduced injury risk (Buchheit et al. 2023). When considering the complexity of sprinting (e.g., kinetics/kinematics, elastic energy transfer, motor behaviour, specific rate of force development etc.), it is impossible to effectively replicate its biomechanics during typical gym‐based resistive exercises (Buchheit et al. 2023; Ward et al. 2024). Hence, regular sprint exposures are necessary as part of a well‐structured strength and conditioning programme for optimal running speed development and hamstring injury prevention (Buchheit et al. 2023; Haugen et al. 2019).

After linear advancing movements, deceleration was the second most prevalent action preceding goals, similar to earlier work (Martínez‐Hernández et al. 2023, 2024). The high occurrence of decelerations herein could plausibly be linked to the idea that players use intense decelerative actions to create separation from defenders, allowing better opportunities to make a successful run, pass, or shot on goal (Ade et al. 2016; Martínez‐Hernández et al. 2023). Indeed, when an offensive player rapidly decelerates, they could disrupt the momentum of a defending player, who may struggle to adjust their own speed and positioning in response (Ade et al. 2016; Martínez‐Hernández et al. 2023). Therefore, improving players' ability to perform high‐intensity and/or repeated decelerations may be crucial for attackers to generate scoring opportunities, and equally for defenders to effectively counter these actions by rapidly closing down space and reacting to offensive moves (Harper et al. 2019). Decelerative actions may create various scoring opportunities, including: (1) running into the penalty box and decelerating to create space for a shot, (2) breaking the opposition's offside trap by breaking just before receiving a pass, or (3) decelerating to create space, then quickly moving into a different area of the opposition's half to receive the ball (Ade et al. 2016; Harper et al. 2024). Research specifically targeting interventions to enhance deceleration capacities remains limited (Harper et al. 2024). Nevertheless, deceleration is an integral component of performance, given that once a player accelerates, they must ultimately reduce speed at varying rates. In this regard, training across all phases (i.e., acceleration, transition, maximal speed, and deceleration) should be emphasized within player preparation. A holistic approach that integrates these phases alongside broader physical qualities (e.g., eccentric strength, reactive strength, and technical execution) is likely to be most beneficial for supporting the complex demands of team sport performance (Daly et al. 2022, 2022; Harper et al. 2024, 2022).

Notably, turning actions occurred at rates similar to decelerations during successful goals from play, a finding in line with earlier work (Martínez‐Hernández and Jones 2024; Martínez‐Hernández et al. 2023, 2024). The complex and integrative nature of turning involves various underpinning factors, such as trunk control, centre of gravity, and neuromuscular qualities, all of which converge with a player's technique execution to determine deceleration, ground contact times and subsequent horizontal and vertical propulsion in the exit direction (Keller et al. 2020). Important to note is that whilst ‘turns’ or ‘cuts’ were coded in this analysis as discrete technical actions, such movements are underpinned by broader change of direction (COD) ability, which reflects the physical capacity to rapidly decelerate and re‐accelerate when altering travel direction (Nygaard Falch et al. 2019) Beyond goal‐scoring situations, the importance of change of direction/turning/cutting ability is further highlighted by evidence that (i) elite players exhibit superior change of direction ability compared to sub‐elite players (Gabbett et al. 2009), and (ii) there are associations between change of direction ability and external training workload performance (Gonçalves et al. 2021). A relatively established literature base suggests that a range of interventions may be used to enhance change of direction ability, such as strength, plyometric, sprint and change of direction/pace training (Nygaard Falch et al. 2019).

In addition to the more common actions like linear advancing, decelerating, and turning, other lateral advancing movements and change in angle runs such as arc runs, cuts, shuffles, and crossovers contributed to the remaining 20% of actions immediately preceding goals. Whilst these movements occur less prevalently, they nevertheless play a significant role in creating opportunities, and it's possible that they may add unpredictability and variation in player movement patterns for instance, which could aid in attacks (Martínez‐Hernández and Jones 2024; Martínez‐Hernández et al. 2023, 2024). As an example, it could be surmised that arc runs could help attackers evade defenders by taking wider angles, whilst shuffles and crossovers could elicit rapid lateral adjustments that allow players to reposition themselves in tight spaces or when under pressure (Ade et al. 2016; Martínez‐Hernández and Jones 2024). Integrating these movement patterns into drills or training games, could provide players with more exposure to the diverse physical and cognitive/tactical demands of goal scoring situations.

In summary, the present findings highlight that high‐intensity actions, such as sprinting, decelerating, changing direction, and lateral movements, are crucial to successful goals during play, underscoring the importance of developing players' neuromuscular qualities. This study presents several limitations that should be acknowledged. Foremost, whilst the modified Bloomfield method has shown strong reliability (Bloomfield et al. 2007; Martínez‐Hernández et al. 2023, 2024), inconsistencies in categorizing movements may still arise, and this should be considered. Nevertheless, current intra‐ and inter‐reliability assessments support earlier work that the method has high reliability. Secondly, the study also did not account for important contextual factors that can influence movement patterns and intensities, such as match score, team tactics, environmental conditions, or the quality of the opposition, variables which could influence the actions leading to goals (Augusto et al. 2021, 2021). Future work may consider to define and conduct analysis of successful turnovers, and other important defensive/offensive actions, in order to provide valuable nuanced insight with practical applications.

## 5 Conclusions

This study provides novel insights into the characterization and intensity of movements that preceded goals from open play in 6 total FIFA World Cups across both men's and women's competitions. Sprints, decelerations, and turns are the most common actions occurring in the lead up to goals from play. Accordingly, practitioners should prepare attackers to execute, and defenders to anticipate/disrupt, intensive sprints, decelerations, and directional changes given their prevalence in the moments preceding goals, regardless of population. The current findings closely cohere with earlier work (Faude et al. 2012; Martínez‐Hernández and Jones 2024; Martínez‐Hernández et al. 2023, 2024), and indicate minimal changes according to competition cycle, aside from a decrease in high intensity actions during the most recent cycle, biological sex or player role. This consistency implicates that the type and relative intensity of actions leading to goals are largely invariant across sex, match intensity, and playing standard, and consequently may translate well to other soccer populations. In applied settings, performance analysts may use these insights to better identify the attacking/defensive behaviours most likely to precede goals, whilst coaches could design drills that rehearse sprints, turns, and decelerations as part of goal‐scoring scenarios. Finally, the large sample in this study, together with the replication of earlier findings in other populations, strengthen confidence that these actions potentially play a causal role in the creation of goal‐scoring opportunities, warranting future inferential work to explore this.

## Ethics Statement

The current study analysed publicly available video footage from 6 FIFA World Cup tournaments to examine movements prior to goals. As the data were fully accessible to the public, institutional ethical approval was deemed to be not required. The analysis adhered to the principles of the Declaration of Helsinki.

## Conflicts of Interest

The authors declare no conflicts of interest.

## Supporting information


Supporting Information S1

## Data Availability

The data underpinning the findings of this research are openly accessible at an open science framework repository, link: https://shorturl.at/OWk2X, thereby promoting transparency and reproducibility. Practitioners or researchers interested in exploring, verifying or reusing this dataset for alternative purposes are encouraged to do so.
