# Supplementary material for: Sprints, Decelerations and Turns Most Commonly Precede Goals in Soccer: Analysis of 6 FIFA World Cups
Source: Eur J Sport Sci. 2025 Nov 23;25(12):e70085. doi: 10.1002/ejsc.70085 (PMC12640733; doi:10.1002/ejsc.70085)
Supplement: Supplementary file 1 — Supporting Information S1 [file EJSC-25-e70085-s001.docx]

**Supplementary Table S1. Interpretation and definitions of movement groups and movements.**

| **Movement Group** | **Definition** |
| --- | --- |
| Linear advancing motion | Actions where a player accelerates or maintains speed in a sagittal plane. |
| Lateral advancing motion | Actions where a player accelerates or maintains speed in a frontal plane. |
| Change in angle run | Actions where a player advancing in a linear direction manoeuvres without, or with very little, loss of speed. |
| Ball blocking | Purposefully driving the lower limb or head to stop a ball or an attacker. |
| Ball striking | Contact made with the ball with the objective of passing or scoring a goal. |
| **Movement** | **Definition** |
| Walk | Moving slowly by stepping.* |
| Jog | Moving at a slow monotonous pace (slower than running, quicker than walking).* |
| Run | Manifest purpose and effort, usually when gaining distance.* |
| Sprint | Maximal effort, rapid motion.* |
| Shuffle | Sideways advancing movement in which head, shoulders and hips face forward while legs and feet do not cross. |
| Crossover | Sideways advancing movement in which head, shoulders and hips face forward while legs and feet cross. |
| Deceleration | To slow down or brake suddenly.** |
| Turn | To rotate while standing, decelerating, or accelerating/sprinting. |
| Cut | Path change of less than 45° involving little or no deceleration to accomplish the task. |
| Arc run | Player (often leaning to one side) moving in a semi-circular direction.* |
| Skip | Moving with small bound-like movements.* |
| Impact | Any intense contact made with another player.* |
| Stand still | More or less stationary or staying in one spot.* |
| Jump | Spring free from the ground or other base by the muscular action of feet and legs.* |
| Land | Entered after jump when contact with ground is made.* |
| Dive | Purposefully and controllably propelling the body rapidly through the air, either feet or head first.* |
| Slide | Purposefully and controllably driving the body along the floor with feet leading the movement. |
| Fall | Descending to the ground.* |
| Get up | Ascending from the ground.* |
| Pass | Any attempt to give the ball to a teammate, entered as contact made with the ball along with how.* |
| Shoot | Any attempt on goal, entered as contact made with the ball along with how.* |

* Definition from Bloomfield et al. (2004)
**Modified definition from Bloomfield et al. (2004). Table taken and included with permission from Martínez-Hernández et al. (2023).

**Supplementary Table S2. Interpretation and definitions of modifiers.** Table taken and included with permission from Martínez-Hernández et al. (2023).

| **Modifier** | **Definition** |
| --- | --- |
| **Direction** |  |
| Forward (linear advancing) | Head, shoulders, and hips all face forward, moving in a forward direction. |
| Forward (deceleration) | Player braking with one or both limbs, stopping body inertia pushing linearly forward. |
| Forward diagonal (linear advancing) | Body turned about 45° left/right; head turned left/right, looking over shoulder; legs facing forward or slightly rotated while advancing.** |
| Forward diagonal (deceleration) | Player braking with one or both limbs and body position turned approximately 45° left/right, stopping body inertia pushing diagonally forward. |
| Backward (linear advancing) | Head, shoulders, and hips all face forward, moving in a backward direction. |
| Backward (deceleration) | Head, shoulders, and hips all face forward, stopping body inertia pushing in a backward direction. |
| 0°–60° turn | Turn ≤ 1/6 circle.* |
| 60°–120° turn | Turn > 1/6 circle and ≤ 1/3 circle.* |
| 120°–180° turn | Turn > 1/3 circle and ≤ 1/2 circle.* |
| 180°–270° turn | Turn > 1/2 circle and ≤ 3/4 circle.* |
| 270°–360° turn | Turn > 3/4 circle and ≤ full circle.* |
| **Intensity** |  |
| Low | Little effort.* |
| Medium | Some to great effort.* |
| High | Maximal effort.* |
| **Ball** |  |
| Yes | Player in possession of the ball. |
| No | Player not in possession of the ball. |

*Definition from Bloomfield et al. (2004)
**Modified definition from Bloomfield et al. (2004). Table taken and included with permission from Martínez-Hernández et al. (2023).

**References**

Bloomfield, J., Polman, R., & O’Donoghue, P. (2004). The ‘Bloomfield Movement Classification’: motion analysis of individual players in dynamic movement sports. *International Journal of Performance Analysis in Sport, 4*(2), 20-31. <https://doi.org/https://doi.org/10.1080/24748668.2004.11868300>

Martínez-Hernández, D., Quinn, M., & Jones, P. (2023). Linear advancing actions followed by deceleration and turn are the most common movements preceding goals in male professional soccer. *Science and medicine in football*, 1-9. <https://doi.org/https://doi.org/10.1080/24733938.2022.2030064>
